# Dr. Arthur's Instrument

**Published:** 1873-05

**Authors:** 


*Dr. Arthur's Instrument.*—Several letters having been received asking for information concerning this instrument, or "dental tool" as it has been designated, we reply that it is a thin corundum wheel, for separating teeth, one-half to one inch in diameter, drawn nearly to an edge on the periphery, either perfectly flat, or slightly beveled toward the centre, and adapted to be rotated between the teeth by a dental engine, either Morrison's, or Green's.

